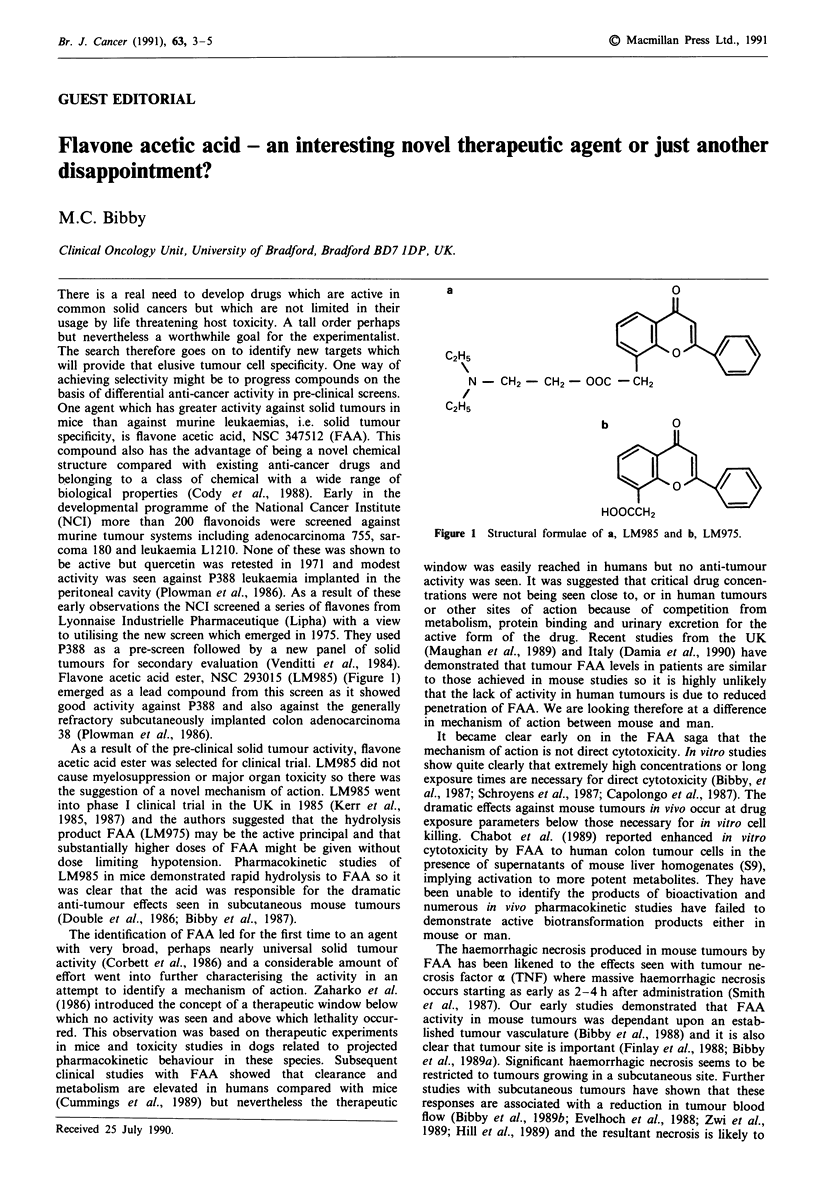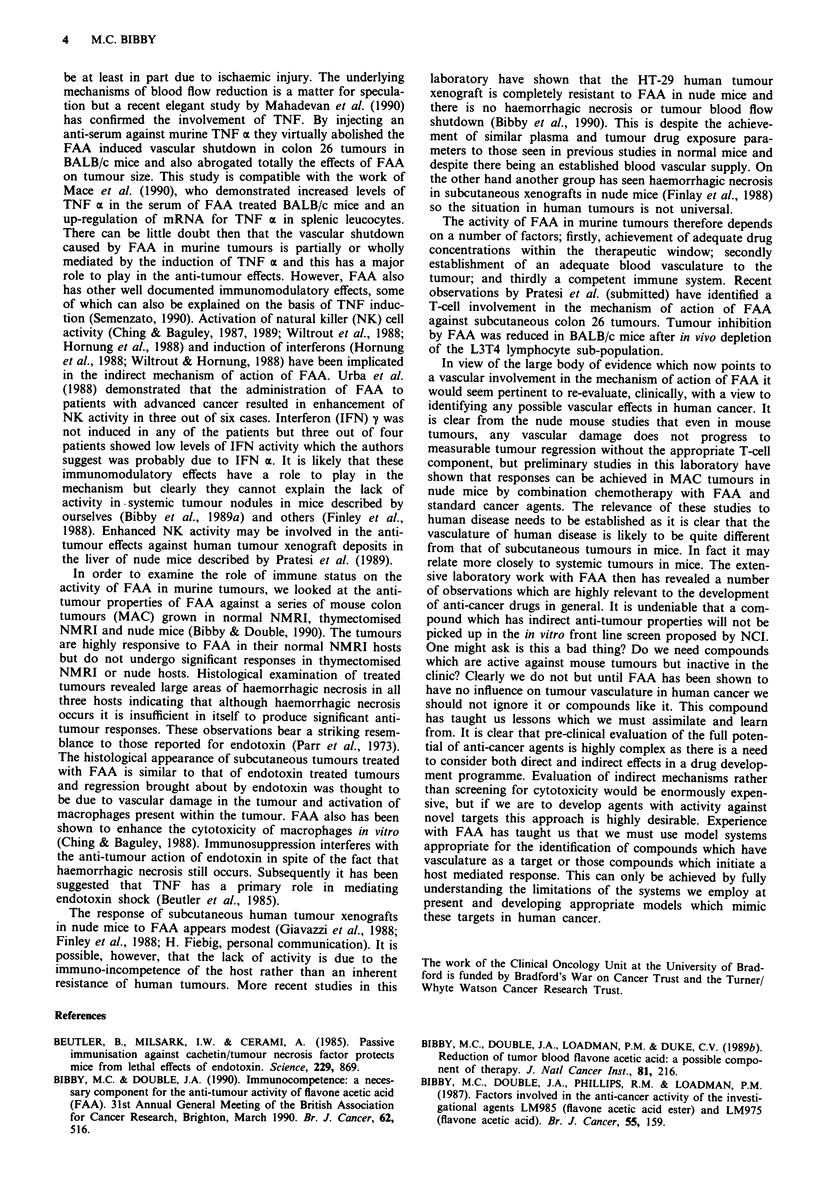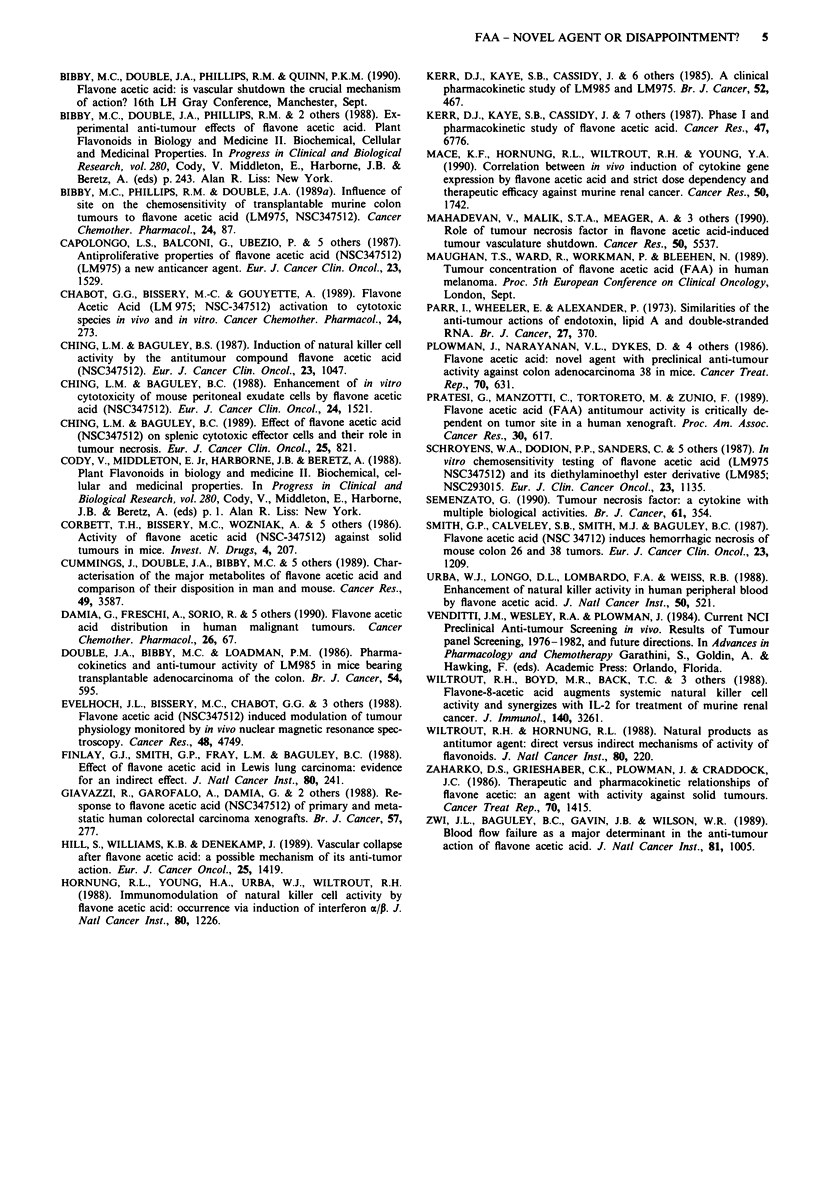# Flavone acetic acid--an interesting novel therapeutic agent or just another disappointment?

**DOI:** 10.1038/bjc.1991.2

**Published:** 1991-01

**Authors:** M. C. Bibby


Br. J. Cancer (1991), 63, 3-5 © Macmillan Press Ltd., 1991

GUEST EDITORIAL

### Flavone acetic acid – an interesting novel therapeutic agent or just another disappointment?

M.C. Bibby

*Clinical Oncology Unit, University of Bradford, Bradford BD7 1DP, UK.*

There is a real need to develop drugs which are active in common solid cancers but which are not limited in their usage by life threatening host toxicity. A tall order perhaps but nevertheless a worthwhile goal for the experimentalist. The search therefore goes on to identify new targets which will provide that elusive tumour cell specificity. One way of achieving selectivity might be to progress compounds on the basis of differential anti-cancer activity in pre-clinical screens. One agent which has greater activity against solid tumours in mice than against murine leukaemias, i.e. solid tumour specificity, is flavone acetic acid, NSC 347512 (FAA). This compound also has the advantage of being a novel chemical structure compared with existing anti-cancer drugs and belonging to a class of chemical with a wide range of biological properties (Cody *et al.*, 1988). Early in the developmental programme of the National Cancer Institute (NCI) more than 200 flavonoids were screened against murine tumour systems including adenocarcinoma 755, sarcoma 180 and leukaemia L1210. None of these was shown to be active but quercetin was retested in 1971 and modest activity was seen against P388 leukaemia implanted in the peritoneal cavity (Plowman *et al.*, 1986). As a result of these early observations the NCI screened a series of flavones from Lyonnaise Industrielle Pharmaceutique (Lipha) with a view to utilising the new screen which emerged in 1975. They used P388 as a pre-screen followed by a new panel of solid tumours for secondary evaluation (Venditti *et al.*, 1984). Flavone acetic acid ester, NSC 293015 (LM985) (Figure 1) emerged as a lead compound from this screen as it showed good activity against P388 and also against the generally refractory subcutaneously implanted colon adenocarcinoma 38 (Plowman *et al.*, 1986).

As a result of the pre-clinical solid tumour activity, flavone acetic acid ester was selected for clinical trial. LM985 did not cause myelosuppression or major organ toxicity so there was the suggestion of a novel mechanism of action. LM985 went into phase I clinical trial in the UK in 1985 (Kerr *et al.*, 1985, 1987) and the authors suggested that the hydrolysis product FAA (LM975) may be the active principal and that substantially higher doses of FAA might be given without dose limiting hypotension. Pharmacokinetic studies of LM985 in mice demonstrated rapid hydrolysis to FAA so it was clear that the acid was responsible for the dramatic anti-tumour effects seen in subcutaneous mouse tumours (Double *et al.*, 1986; Bibby *et al.*, 1987).

The identification of FAA led for the first time to an agent with very broad, perhaps nearly universal solid tumour activity (Corbett *et al.*, 1986) and a considerable amount of effort went into further characterising the activity in an attempt to identify a mechanism of action. Zaharko *et al.* (1986) introduced the concept of a therapeutic window below which no activity was seen and above which lethality occurred. This observation was based on therapeutic experiments in mice and toxicity studies in dogs related to projected pharmacokinetic behaviour in these species. Subsequent clinical studies with FAA showed that clearance and metabolism are elevated in humans compared with mice (Cummings *et al.*, 1989) but nevertheless the therapeutic

Received 25 July 1990.

**Figure 1** Structural formulae of **a**, LM985 and **b**, LM975.

window was easily reached in humans but no anti-tumour activity was seen. It was suggested that critical drug concentrations were not being seen close to, or in human tumours or other sites of action because of competition from metabolism, protein binding and urinary excretion for the active form of the drug. Recent studies from the UK (Maughan *et al.*, 1989) and Italy (Damia *et al.*, 1990) have demonstrated that tumour FAA levels in patients are similar to those achieved in mouse studies so it is highly unlikely that the lack of activity in human tumours is due to reduced penetration of FAA. We are looking therefore at a difference in mechanism of action between mouse and man.

It became clear early on in the FAA saga that the mechanism of action is not direct cytotoxicity. *In vitro* studies show quite clearly that extremely high concentrations or long exposure times are necessary for direct cytotoxicity (Bibby, *et al.*, 1987; Schroyens *et al.*, 1987; Capolongo *et al.*, 1987). The dramatic effects against mouse tumours *in vivo* occur at drug exposure parameters below those necessary for *in vitro* cell killing. Chabot *et al.* (1989) reported enhanced *in vitro* cytotoxicity by FAA to human colon tumour cells in the presence of supernatants of mouse liver homogenates (S9), implying activation to more potent metabolites. They have been unable to identify the products of bioactivation and numerous *in vivo* pharmacokinetic studies have failed to demonstrate active biotransformation products either in mouse or man.

The haemorrhagic necrosis produced in mouse tumours by FAA has been likened to the effects seen with tumour necrosis factor α (TNF) where massive haemorrhagic necrosis occurs starting as early as 2–4 h after administration (Smith *et al.*, 1987). Our early studies demonstrated that FAA activity in mouse tumours was dependant upon an established tumour vasculature (Bibby *et al.*, 1988) and it is also clear that tumour site is important (Finlay *et al.*, 1988; Bibby *et al.*, 1989*a*). Significant haemorrhagic necrosis seems to be restricted to tumours growing in a subcutaneous site. Further studies with subcutaneous tumours have shown that these responses are associated with a reduction in tumour blood flow (Bibby *et al.*, 1989*b*; Evelhoch *et al.*, 1988; Zwi *et al.*, 1989; Hill *et al.*, 1989) and the resultant necrosis is likely to be at least in part due to ischaemic injury. The underlying mechanisms of blood flow reduction is a matter for speculation but a recent elegant study by Mahadevan *et al.* (1990) has confirmed the involvement of TNF. By injecting an anti-serum against murine TNF α they virtually abolished the FAA induced vascular shutdown in colon 26 tumours in BALB/c mice and also abrogated totally the effects of FAA on tumour size. This study is compatible with the work of Mace *et al.* (1990), who demonstrated increased levels of TNF α in the serum of FAA treated BALB/c mice and an up-regulation of mRNA for TNF α in splenic leucocytes. There can be little doubt then that the vascular shutdown caused by FAA in murine tumours is partially or wholly mediated by the induction of TNF α and this has a major role to play in the anti-tumour effects. However, FAA also has other well documented immunomodulatory effects, some of which can also be explained on the basis of TNF induction (Semenzato, 1990). Activation of natural killer (NK) cell activity (Ching & Baguley, 1987, 1989; Wiltrout *et al.*, 1988; Hornung *et al.*, 1988) and induction of interferons (Hornung *et al.*, 1988; Wiltrout & Hornung, 1988) have been implicated in the indirect mechanism of action of FAA. Urba *et al.* (1988) demonstrated that the administration of FAA to patients with advanced cancer resulted in enhancement of NK activity in three out of six cases. Interferon (IFN) γ was not induced in any of the patients but three out of four patients showed low levels of IFN activity which the authors suggest was probably due to IFN α. It is likely that these immunomodulatory effects have a role to play in the mechanism but clearly they cannot explain the lack of activity in systemic tumour nodules in mice described by ourselves (Bibby *et al.*, 1989*a*) and others (Finley *et al.*, 1988). Enhanced NK activity may be involved in the anti-tumour effects against human tumour xenograft deposits in the liver of nude mice described by Pratesi *et al.* (1989).

In order to examine the role of immune status on the activity of FAA in murine tumours, we looked at the anti-tumour properties of FAA against a series of mouse colon tumours (MAC) grown in normal NMRI, thymectomised NMRI and nude mice (Bibby & Double, 1990). The tumours are highly responsive to FAA in their normal NMRI hosts but do not undergo significant responses in thymectomised NMRI or nude hosts. Histological examination of treated tumours revealed large areas of haemorrhagic necrosis in all three hosts indicating that although haemorrhagic necrosis occurs it is insufficient in itself to produce significant anti-tumour responses. These observations bear a striking resemblance to those reported for endotoxin (Parr *et al.*, 1973). The histological appearance of subcutaneous tumours treated with FAA is similar to that of endotoxin treated tumours and regression brought about by endotoxin was thought to be due to vascular damage in the tumour and activation of macrophages present within the tumour. FAA also has been shown to enhance the cytotoxicity of macrophages *in vitro* (Ching & Baguley, 1988). Immunosuppression interferes with the anti-tumour action of endotoxin in spite of the fact that haemorrhagic necrosis still occurs. Subsequently it has been suggested that TNF has a primary role in mediating endotoxin shock (Beutler *et al.*, 1985).

The response of subcutaneous human tumour xenografts in nude mice to FAA appears modest (Giavazzi *et al.*, 1988; Finley *et al.*, 1988; H. Fiebig, personal communication). It is possible, however, that the lack of activity is due to the immuno-incompetence of the host rather than an inherent resistance of human tumours. More recent studies in this laboratory have shown that the HT-29 human tumour xenograft is completely resistant to FAA in nude mice and there is no haemorrhagic necrosis or tumour blood flow shutdown (Bibby *et al.*, 1990). This is despite the achievement of similar plasma and tumour drug exposure parameters to those seen in previous studies in normal mice and despite there being an established blood vascular supply. On the other hand another group has seen haemorrhagic necrosis in subcutaneous xenografts in nude mice (Finlay *et al.*, 1988) so the situation in human tumours is not universal.

The activity of FAA in murine tumours therefore depends on a number of factors; firstly, achievement of adequate drug concentrations within the therapeutic window; secondly establishment of an adequate blood vasculature to the tumour; and thirdly a competent immune system. Recent observations by Pratesi *et al.* (submitted) have identified a T-cell involvement in the mechanism of action of FAA against subcutaneous colon 26 tumours. Tumour inhibition by FAA was reduced in BALB/c mice after *in vivo* depletion of the L3T4 lymphocyte sub-population.

In view of the large body of evidence which now points to a vascular involvement in the mechanism of action of FAA it would seem pertinent to re-evaluate, clinically, with a view to identifying any possible vascular effects in human cancer. It is clear from the nude mouse studies that even in mouse tumours, any vascular damage does not progress to measurable tumour regression without the appropriate T-cell component, but preliminary studies in this laboratory have shown that responses can be achieved in MAC tumours in nude mice by combination chemotherapy with FAA and standard cancer agents. The relevance of these studies to human disease needs to be established as it is clear that the vasculature of human disease is likely to be quite different from that of subcutaneous tumours in mice. In fact it may relate more closely to systemic tumours in mice. The extensive laboratory work with FAA then has revealed a number of observations which are highly relevant to the development of anti-cancer drugs in general. It is undeniable that a compound which has indirect anti-tumour properties will not be picked up in the *in vitro* front line screen proposed by NCI. One might ask is this a bad thing? Do we need compounds which are active against mouse tumours but inactive in the clinic? Clearly we do not but until FAA has been shown to have no influence on tumour vasculature in human cancer we should not ignore it or compounds like it. This compound has taught us lessons which we must assimilate and learn from. It is clear that pre-clinical evaluation of the full potential of anti-cancer agents is highly complex as there is a need to consider both direct and indirect effects in a drug development programme. Evaluation of indirect mechanisms rather than screening for cytotoxicity would be enormously expensive, but if we are to develop agents with activity against novel targets this approach is highly desirable. Experience with FAA has taught us that we must use model systems appropriate for the identification of compounds which have vasculature as a target or those compounds which initiate a host mediated response. This can only be achieved by fully understanding the limitations of the systems we employ at present and developing appropriate models which mimic these targets in human cancer.

The work of the Clinical Oncology Unit at the University of Bradford is funded by Bradford's War on Cancer Trust and the Turner/Whyte Watson Cancer Research Trust.

**References**

BEUTLER, B., MILSARK, I.W. & CERAMI, A. (1985). Passive immunisation against cachetin/tumour necrosis factor protects mice from lethal effects of endotoxin. *Science*, **229**, 869.

BIBBY, M.C. & DOUBLE, J.A. (1990). Immunocompetence: a necessary component for the anti-tumour activity of flavone acetic acid (FAA). 31st Annual General Meeting of the British Association for Cancer Research, Brighton, March 1990. *Br. J. Cancer*, **62**, 516.

BIBBY, M.C., DOUBLE, J.A., LOADMAN, P.M. & DUKE, C.V. (1989*b*). Reduction of tumor blood flavone acetic acid: a possible component of therapy. *J. Natl Cancer Inst.*, **81**, 216.

BIBBY, M.C., DOUBLE, J.A., PHILLIPS, R.M. & LOADMAN, P.M. (1987). Factors involved in the anti-cancer activity of the investigational agents LM985 (flavone acetic acid ester) and LM975 (flavone acetic acid). *Br. J. Cancer*, **55**, 159.

BIBBY, M.C., DOUBLE, J.A., PHILLIPS, R.M. & QUINN, P.K.M. (1990). Flavone acetic acid: is vascular shutdown the crucial mechanism of action? 16th LH Gray Conference, Manchester, Sept.

BIBBY, M.C., DOUBLE, J.A., PHILLIPS, R.M. & 2 others (1988). Experimental anti-tumour effects of flavone acetic acid. Plant Flavonoids in Biology and Medicine II. Biochemical, Cellular and Medicinal Properties. In *Progress in Clinical and Biological Research, vol. 280,* Cody, V. Middleton, E., Harborne, J.B. & Beretz, A. (eds) p. 243. Alan R. Liss: New York.

BIBBY, M.C., PHILLIPS, R.M. & DOUBLE, J.A. (1989*a*). Influence of site on the chemosensitivity of transplantable murine colon tumours to flavone acetic acid (LM975, NSC347512). *Cancer Chemother. Pharmacol.*, **24,** 87.

CAPOLONGO, L.S., BALCONI, G., UBEZIO, P. & 5 others (1987). Antiproliferative properties of flavone acetic acid (NSC347512) (LM975) a new anticancer agent. *Eur. J. Cancer Clin. Oncol.*, **23,** 1529.

CHABOT, G.G., BISSERY, M.-C. & GOUYETTE, A. (1989). Flavone Acetic Acid (LM 975; NSC-347512) activation to cytotoxic species *in vivo* and *in vitro*. *Cancer Chemother. Pharmacol.*, **24,** 273.

CHING, L.M. & BAGULEY, B.S. (1987). Induction of natural killer cell activity by the antitumour compound flavone acetic acid (NSC347512). *Eur. J. Cancer Clin. Oncol.*, **23,** 1047.

CHING, L.M. & BAGULEY, B.C. (1988). Enhancement of *in vitro* cytotoxicity of mouse peritoneal exudate cells by flavone acetic acid (NSC347512). *Eur. J. Cancer Clin. Oncol.*, **24,** 1521.

CHING, L.M. & BAGULEY, B.C. (1989). Effect of flavone acetic acid (NSC347512) on splenic cytotoxic effector cells and their role in tumour necrosis. *Eur. J. Cancer Clin. Oncol.*, **25,** 821.

CODY, V., MIDDLETON, E. Jr, HARBORNE, J.B. & BERETZ, A. (1988). Plant Flavonoids in biology and medicine II. Biochemical, cellular and medicinal properties. In *Progress in Clinical and Biological Research, vol. 280,* Cody, V., Middleton, E., Harborne, J.B. & Beretz, A. (eds) p. 1. Alan R. Liss: New York.

CORBETT, T.H., BISSERY, M.C., WOZNIAK, A. & 5 others (1986). Activity of flavone acetic acid (NSC-347512) against solid tumours in mice. *Invest. N. Drugs*, **4,** 207.

CUMMINGS, J., DOUBLE, J.A., BIBBY, M.C. & 5 others (1989). Characterisation of the major metabolites of flavone acetic acid and comparison of their disposition in man and mouse. *Cancer Res.*, **49,** 3587.

DAMIA, G., FRESCHI, A., SORIO, R. & 5 others (1990). Flavone acetic acid distribution in human malignant tumours. *Cancer Chemother. Pharmacol.*, **26,** 67.

DOUBLE, J.A., BIBBY, M.C. & LOADMAN, P.M. (1986). Pharmacokinetics and anti-tumour activity of LM985 in mice bearing transplantable adenocarcinoma of the colon. *Br. J. Cancer*, **54,** 595.

EVELHOCH, J.L., BISSERY, M.C., CHABOT, G.G. & 3 others (1988). Flavone acetic acid (NSC347512) induced modulation of tumour physiology monitored by *in vivo* nuclear magnetic resonance spectroscopy. *Cancer Res.*, **48,** 4749.

FINLAY, G.J., SMITH, G.P., FRAY, L.M. & BAGULEY, B.C. (1988). Effect of flavone acetic acid in Lewis lung carcinoma: evidence for an indirect effect. *J. Natl Cancer Inst.*, **80,** 241.

GIAVAZZI, R., GAROFALO, A., DAMIA, G. & 2 others (1988). Response to flavone acetic acid (NSC347512) of primary and metastatic human colorectal carcinoma xenografts. *Br. J. Cancer*, **57,** 277.

HILL, S., WILLIAMS, K.B. & DENEKAMP, J. (1989). Vascular collapse after flavone acetic acid: a possible mechanism of its anti-tumor action. *Eur. J. Cancer Oncol.*, **25,** 1419.

HORNUNG, R.L., YOUNG, H.A., URBA, W.J., WILTROUT, R.H. (1988). Immunomodulation of natural killer cell activity by flavone acetic acid: occurrence via induction of interferon $\alpha/\beta$. *J. Natl Cancer Inst.*, **80,** 1226.

KERR, D.J., KAYE, S.B., CASSIDY, J. & 6 others (1985). A clinical pharmacokinetic study of LM985 and LM975. *Br. J. Cancer*, **52,** 467.

KERR, D.J., KAYE, S.B., CASSIDY, J. & 7 others (1987). Phase I and pharmacokinetic study of flavone acetic acid. *Cancer Res.*, **47,** 6776.

MACE, K.F., HORNUNG, R.L., WILTROUT, R.H. & YOUNG, Y.A. (1990). Correlation between *in vivo* induction of cytokine gene expression by flavone acetic acid and strict dose dependency and therapeutic efficacy against murine renal cancer. *Cancer Res.*, **50,** 1742.

MAHADEVAN, V., MALIK, S.T.A., MEAGER, A. & 3 others (1990). Role of tumour necrosis factor in flavone acetic acid-induced tumour vasculature shutdown. *Cancer Res.*, **50,** 5537.

MAUGHAN, T.S., WARD, R., WORKMAN, P. & BLEEHEN, N. (1989). Tumour concentration of flavone acetic acid (FAA) in human melanoma. *Proc. 5th European Conference on Clinical Oncology*, London, Sept.

PARR, I., WHEELER, E. & ALEXANDER, P. (1973). Similarities of the anti-tumour actions of endotoxin, lipid A and double-stranded RNA. *Br. J. Cancer*, **27,** 370.

PLOWMAN, J., NARAYANAN, V.L., DYKES, D. & 4 others (1986). Flavone acetic acid: novel agent with preclinical anti-tumour activity against colon adenocarcinoma 38 in mice. *Cancer Treat. Rep.*, **70,** 631.

PRATESI, G., MANZOTTI, C., TORTORETO, M. & ZUNIO, F. (1989). Flavone acetic acid (FAA) antitumour activity is critically dependent on tumor site in a human xenograft. *Proc. Am. Assoc. Cancer Res.*, **30,** 617.

SCHROYENS, W.A., DODION, P.P., SANDERS, C. & 5 others (1987). *In vitro* chemosensitivity testing of flavone acetic acid (LM975 NSC347512) and its diethylaminoethyl ester derivative (LM985; NSC293015. *Eur. J. Clin. Cancer Oncol.*, **23,** 1135.

SEMENZATO, G. (1990). Tumour necrosis factor: a cytokine with multiple biological activities. *Br. J. Cancer*, **61,** 354.

SMITH, G.P., CALVELEY, S.B., SMITH, M.J. & BAGULEY, B.C. (1987). Flavone acetic acid (NSC 34712) induces hemorrhagic necrosis of mouse colon 26 and 38 tumors. *Eur. J. Cancer Clin. Oncol.*, **23,** 1209.

URBA, W.J., LONGO, D.L., LOMBARDO, F.A. & WEISS, R.B. (1988). Enhancement of natural killer activity in human peripheral blood by flavone acetic acid. *J. Natl Cancer Inst.*, **50,** 521.

VENDITTI, J.M., WESLEY, R.A. & PLOWMAN, J. (1984). Current NCI Preclinical Anti-tumour Screening *in vivo*. Results of Tumour panel Screening, 1976–1982, and future directions. In *Advances in Pharmacology and Chemotherapy* Garathini, S., Goldin, A. & Hawking, F. (eds). Academic Press: Orlando, Florida.

WILTROUT, R.H., BOYD, M.R., BACK, T.C. & 3 others (1988). Flavone-8-acetic acid augments systemic natural killer cell activity and synergizes with IL-2 for treatment of murine renal cancer. *J. Immunol.*, **140,** 3261.

WILTROUT, R.H. & HORNUNG, R.L. (1988). Natural products as antitumor agent: direct versus indirect mechanisms of activity of flavonoids. *J. Natl Cancer Inst.*, **80,** 220.

ZAHARKO, D.S., GRIESHABER, C.K., PLOWMAN, J. & CRADDOCK, J.C. (1986). Therapeutic and pharmacokinetic relationships of flavone acetic: an agent with activity against solid tumours. *Cancer Treat Rep.*, **70,** 1415.

ZWI, J.L., BAGULEY, B.C., GAVIN, J.B. & WILSON, W.R. (1989). Blood flow failure as a major determinant in the anti-tumour action of flavone acetic acid. *J. Natl Cancer Inst.*, **81,** 1005.